# Supplementary material for: Characterization of Latin American migrants at risk for Trypanosoma cruzi infection in a non-endemic setting. Insights into initial evaluation of cardiac and digestive involvement
Source: PLoS Negl Trop Dis. 2023 Jul 13;17(7):e0011330. doi: 10.1371/journal.pntd.0011330 (PMC10343076; doi:10.1371/journal.pntd.0011330)
Supplement: Attachment — Submitted filename: ResponseToReviewers.docx [file pntd.0011330.s002.docx]

Dear Reviewers and editorial team,

We would like to express our sincere gratitude for the time and effort you have invested in reviewing our paper. Your feedback and suggestions have been extremely helpful in improving the overall quality of our work.

We have carefully considered all your comments and have made significant changes to the manuscript in accordance with your suggestions. Additionally, we have also considered other few suggestions that we did not implement and have provided explanations for why we have chosen not to do so.

Once again, we would like to thank you for your input. We believe that your contributions have greatly enhanced the overall quality of our manuscript. We hope that this new version meets the standards of the journal and is suitable for publication.

A version with the specific changes highlighted in yellow has been submitted, and a clean version without the highlighted changes is also available.

Please find below the details of the changes and considerations made point by pint.

Sincerely,

Pedro Laynez-Roldán

## Introduction

As suggested, the introduction of the paper was significantly shortened from 100 to 55 lines.

Regarding the suggestions from the reviewer #2:

- **Line 90 (former 92):** The sentence has been changed to indicate that the infections present were mostly acquired by vectorial route, this not necessarily being the majority route of transmission today.
- **Line 91 (former 93-94):** the sentence on metacyclic trypomastigotes has been removed.
- **Former line 94:** the sentence on *T infestans* was eliminated.
- **Line 92 (former line 96):** *relative importance of oral vs vectorial transmission.* Since this is a study focused on patients in a non-endemic area, we have not considered it relevant to expand the information on the relative importance of each form of transmission in an endemic area. Therefore, we maintain that it is a relevant form given the clinical relevance due to the severity of its presentation in a high percentage of cases, without establishing the current relative importance of each of the mechanisms.
- **Line 99 (former 104-105):** *rates of transfusion transmitted infection.*
  By the same logic as in the previous comment, transmission rates by transfusion are not specified as it is exceptional in non-endemic setting.
- **Lines 91-93 (previously on 106-107):** the sentence regarding the endemic area was rephrased considering that Latin America is not a purely geographical but rather cultural concept, containing countries both is South, Central and North America (México being the case). Thus, a specific mention to the US was made since it would be left out of the LA concept.
- **Former line 110:** the phrase containing ‘orography’ was deleted.
- **Former line 125:** we agree with the notion *that “infection in the GI tract most significantly involves the autonomic ganglia”,* but we have not delved into these details as it is not the aim of this manuscript.
- **Former lines 144 and 149:** the section on diagnosis has been significantly shortened so these phrases do not appear in the new version of the manuscript

## Methods

We would like to point out that we respectfully disagree with Reviewer #2 in the notion of this manuscript not tackling a specific research question and hence considering it something other than an original research paper. An opinion that Reviewer #1 does not share either. We indeed have the research questions corresponding to a descriptive study: what are the demographic and epidemiological characteristics of the population at risk in Barcelona (for this reason, a description of the demographic and epidemiological characteristics was made), and what is the clinical profile of the patient with *T. cruzi* infection in the first step of diagnosis (that is why we have analyzed the recorded results of the tests).

However, we agree that the length of some sections of the text such as the introduction may have averted the attention. Thus, we have made changes for clarity in this new version of the manuscript.

## Results

Some changes have been made, we would like to thank the reviewers for spotting some mistakes and inaccuracies:

- Percentages have been rounded to one decimal figure, see changes throughout the manuscript.
- The adjustment variables were not specified because they are the same as those in the table. We have changed the name of the table to “multivariate model”, to make it clearer. (table 2, line 242).
- OR of Bolivia was corrected (table 2)
- Map 3 was removed
- Venn diagram was better explained for clarity (line 219-223, Fig 1). The most remarkable results have been included in the main text (line 227-231).

## Discussion and conclusions

As suggested, a sentence has been introduced to clarify that although the sensitivity of the ECG may be limited, it is an accessible and low-cost test capable of detecting potentially serious abnormalities. Lines 339-341.

As suggested, we expanded the citations of studies outside of Europe in order to expand the discussion around non-endemic settings (line 291-292).

As advised, the overall length of the discussion was reduced from 106 to 93 lines.

The sentence regarding disease progression and treatment was rephrased (line 332-334) and more citations were added.

- *Trypanosoma cruzi* infection vs Chagas disease

Thank you for this suggestion. We strongly agree that it is worth distinguishing both situations. Thus, we have careful reworded some parts of the text to distinguish. Errors throughout the manuscript have been corrected.

- ECG and Echocardiogram

We appreciate the perspective, but we respectfully hold a different view on the relationship between diastolic dysfunction and Chagas Disease. While diastolic dysfunction is commonly observed in the general population and individuals with hypertension, several studies indicate that it could also play a significant role in the progression of Chagas Disease.

For example, a study performed on over 900 patients with CD it was found that *“Diastolic function is prematurely affected in chronic Chagas disease”*, with *“increasing stages of severity of the clinical form associated with progressive deterioration of diastolic function”*. These observations remained even after excluding patients with hypertension as a comorbidity (Siciliano, A. P. d. R. V., et al. (2006). "Padrão da função diastólica e relação com a gravidade da forma clínica em 902 pacientes na fase crônica da doença de Chagas." Rev. SOCERJ 19(1): 74-83).
Moreover, in a recent work (Echeverria, L. E., et al. (2021). "Cardiovascular Biomarkers and Diastolic Dysfunction in Patients With Chronic Chagas Cardiomyopathy." Frontiers in Cardiovascular Medicine), it is stated that *“diastolic dysfunction (DD) in the setting of CCM has also been associated with a worse clinical status and a higher incidence of adverse cardiovascular outcomes. This highlights the importance of making a prompt diagnosis of DD, which is usually achieved by echocardiography”.*

Regarding the prevalence of diastolic dysfunction in the general population, Kuznetsova et al (2009) reported a prevalence of 27.3% among all participants in their study, but only 6% among those who were less than 49 years old (Table 3 of the cited article). In our study, the prevalence of diastolic dysfunction was 17.9% (Table 4), but it's worth noting that more than 85% of our participants were less than 49 years old (Table 1). We would also like to clarify that among the 16 individuals with echocardiogram disorders and normal ECG, 9 had diastolic dysfunction, which accounts for 56.3%, rather than 90% (Lines 271-272). We have included this clarification in our manuscript (Line 272).

However, we are aware that this is a subject under discussion among the scientific community and, therefore, we have not been categorical, but have explained the reasons that lead us to consider diastolic dysfunction as Chagasic Cardiomyopathy.

We agree that techniques more sensitive than ECG would risk finding minor abnormalities in people with *T. cruzi* infection. However, this does not mean that those abnormalities should neither be disregarded nor taken as a fatal result that would lead to drastic clinical decisions.
Indeed, in the reference provided by reviewer #1, authors come to the same conclusion as we do: “ECG and conventional ECHO should be used routinely as standard cardiac tests for newly diagnosed cases of Chagas disease”. Moreover, the notion that patients with “indeterminate” form of the disease who have subtle abnormalities do not progress in longitudinal studies is currently under question, as longer longitudinal studies are being performed with the aid of electronic clinical records.
The combination of ECG and TTE in the first assessment of patients diagnosed with *T. cruzi* infection allows a better assessment of patients who need closer follow-up. Certainly, this strategy can only be followed in places with access to the techniques, but be part of a resource optimization strategy if it is properly interpreted.
As for the risk of “labelling” someone as having CCC, it is true that there is a lot of work to be done against the stigma of the disease in general. But the responsibility for the interpretation of the results for the patients remains with the clinician. Thus, the patient can be informed that an alteration such as mild diastolic dysfunction does not affect eligibility for trypanocide treatment, and that follow-up will be offered according to the risk of progression.

- Comorbidities

Since comorbidities were not specifically addressed in this study, it was added as a limitation (line 372). We agree that they will become increasingly important with the aging of migrant population, but we estimate the impact on the overall findings of this study would probably be minimal due to the mean and median age of the participants.

- Follow up

Indeed, we agree with the notion of the importance of longitudinal follow up of cohorts such as the one we describe in order to answer many of the questions about progression of disease outside of endemic areas. Hence, we added a phrase in this regard (lines 382-383).
