# Supplementary material for: Characterization of Latin American migrants at risk for Trypanosoma cruzi infection in a non-endemic setting. Insights into initial evaluation of cardiac and digestive involvement
Source: PLoS Negl Trop Dis. 2023 Jul 13;17(7):e0011330. doi: 10.1371/journal.pntd.0011330 (PMC10343076; doi:10.1371/journal.pntd.0011330)
Supplement: Attachment — Submitted filename: cover letter. response minor revisions.docx [file pntd.0011330.s004.docx]

Dear Editors,

Thank you for taking the time to review our manuscript. We have carefully considered your comments and have implemented the proposed changes, which we believe have improved the structure and writing of our manuscript.

Regarding the body of the manuscript, we have accepted the changes proposed. Below, we list all the changes made from the previous version:

- Abstract:
  - Corrected and improved the language.
  - Restructured the “Background” section of the abstract.
  - Added absolute numbers in the Result section of the abstract (not just percentages).
  - Rewrote some sentences in the conclusion of the abstract for clarity (Lines 56-61)
- Introduction:
  - Corrected language to clarify.
  - Summarized and reduced details on transmission methods.
  - Removed section numbering.
- Materials and Methods:
  - Improved language to enhance comprehension in the “Design, setting, and recruitment” section.
  - Corrected an error regarding inclusion criteria, which did not include individuals at risk who were not originally from endemic countries (Line 146)
- Results:
  - Corrected language issues.
  - Removed section numbering.
- Discussion:
  - Added a clarifying sentence on the limitations of not including comorbidities.

Additionally, we have italicized some scientific names that were in normal font, and removed section numbering of the general sections.

We have also addressed the additional instruction you raised by:

- Uploading the figures to the PACE digital diagnostic tool. We have resized and resubmitted Figure 2 as suggested.
- The data supporting the study have been deposited in a repository and are referenced in the manuscript (<https://doi.org/10.34810/data223>). We have also added supplementary material to facilitate the reproducibility of Figure 1.
- Regarding laboratory protocols, we do not have a specific protocol at our center
- We have reviewed the reference list, and to our knowledge, there are no withdrawn or retracted articles. However, we have corrected a mistake on the URL of reference 15th and the first author of reference 45th.

Thank you again for your contributions to this manuscript. We hope that the changes we have made meet your expectations for publication. If there are any further changes or suggestions you would like to provide, please let us know.

Thank you for your time and consideration,

Best regards,

Pedro Laynez-Roldán and co-authors
